# Sports activity limitation during the COVID-19 pandemic in young Italian athletes: impact on mental health in children, adolescents, and young adults

**DOI:** 10.3389/fpubh.2023.1237443

**Published:** 2023-08-10

**Authors:** Elisa Tomezzoli, Oriana D'Ecclesiis, Sara Raimondi, Gabriella Pravettoni, Giulio Cammarata, Giovanna Testa, Federica Bellerba, Patrizia Gnagnarella, Maria Luisa Iannuzzo, Alessandro Sartorio, Clementina Sasso, Dorotea Ricci, Nicoletta Marazzi, Federica Galli, Sara Gandini

**Affiliations:** ^1^Applied Research Division for Cognitive and Psychological Science, IEO, European Institute of Oncology Istituto di Ricovero e Cura a Carattere Scientifico (IRCCS), Milan, Italy; ^2^Molecular and Pharmaco-Epidemiology Unit, Department of Experimental Oncology, IEO, European Institute of Oncology Istituto di Ricovero e Cura a Carattere Scientifico (IRCCS), Milan, Italy; ^3^Independent Researcher, Milan, Italy; ^4^Division of Epidemiology and Biostatistics, IEO, European Institute of Oncology Istituto di Ricovero e Cura a Carattere Scientifico (IRCCS), Milan, Italy; ^5^AULSS 9 Scaligera, Dipartimento di Prevenzione, UOC Medicina Legale, Verona, Italy; ^6^Experimental Laboratory for Auxo-endocrinological Research, Istituto Auxologico Italiano, Istituto di Ricovero e Cura a Carattere Scientifico (IRCCS), Milan, Italy; ^7^Istituto Nazionale di Astrofisica (INAF)-Capodimonte Astronomical Observatory, Naples, Italy; ^8^ARES-ODV Associazione Regionale Emergenza Sanitaria, Ancona, Italy; ^9^Department of Dynamic and Clinical Psychology, and Health Studies, Faculty of Medicine and Psychology, Sapienza University of Rome, Rome, Italy

**Keywords:** COVID-19, preventive measures, physical activity, mental health, children, adolescents, young adults

## Abstract

**Introduction:**

The closure of sports centres was implemented as a preventive measure to mitigate the transmission of SARS-CoV-2. Given the observed global decline in physical activity and concurrent rise in sedentary behaviour, even among younger age groups, a retrospective cross-sectional study was undertaken to evaluate the effects of this measure on mental health in children, adolescents, and young adults during the initial phases of the COVID-19 pandemic.

**Methods:**

A total of 1,717 non-professional athletes (age range: 6–25; 53.9% males, 44.6% females) completed an online questionnaire including widely used and validated measures for mental health assessment (SDQ and PGWB-S) and questions regarding sociodemographic characteristics (such as gender), physical activity, and screen time. The association between mental health and sociodemographic characteristics, physical activity, and screen time was evaluated by using univariate and multivariable logistic regression models.

**Results:**

In children and adolescents, the incidence of psychological difficulties was associated with not being physically active (OR = 1.49; 95% CI: 1.09, 2.07; *p* = 0.015). Engaging in physical activity during the period of closures, particularly if more than twice a week, was significantly associated with less psychological difficulties for children/adolescents (OR = 0.54; 95% CI: 0.35, 0.82; *p* = 0.004) and psychological symptoms (i.e., psychological well-being lower than the median) for youth/young adults (OR = 0.25; 95% CI: 0.14, 0.45; *p* < 0.001). More psychological difficulties were also found in males for children and adolescents (OR = 1.37; 95% CI: 1.06, 1.79; *p* = 0.018). However, young adult males showed less psychological symptoms than females (OR = 0.35; 95% CI: 0.22, 0.55; *p* = 0.001). Additionally, a greater amount of screen time was associated with a higher incidence of psychological symptoms in the whole sample.

**Conclusions:**

Our results confirm the positive impact of physical activity on mental health during the COVID-19 pandemic among younger age groups. They also provide valuable insights into the risk-benefit relationship of interrupting sports activities as a preventive measure for infectious diseases.

## Introduction

Coronavirus disease 2019 (COVID-19), caused by the SARS-CoV-2 infection, was first identified in China in December 2019 (1). Since the virus started spreading steadily in several countries in all continents, the World Health Organisation (WHO) declared a pandemic in March 2020. Having it posed a serious threat to public health worldwide, many governments introduced lockdowns to reduce the spread of the virus. Since transmission of SARS-CoV-2 is mainly related to direct exposure to aerosolised respiratory particles (2), it was suggested that their spread could be enhanced by high-intensity exercise (3). Indeed, during the different pandemic waves, shutdown of sports activities, centres, and clubs was included among other preventive measures.

However, it is well-known that physical activity (PA) improves physical and psychological well-being at any age. Specifically, PA is beneficial for the preservation of the immune system, cardiorespiratory and muscular fitness, cardiometabolic health, and bone health (4, 5). It is therefore crucial in preventing infections, cardiovascular diseases, type-2 diabetes, and certain types of cancer (6). As for the psychological aspect, PA entails release of endorphins and reduction in the levels of stress hormones such as cortisol and adrenaline, thus being associated with lower levels of stress, anxiety, and depression (7–11).

Given that risk factors for severe COVID-19 include heart and respiratory problems, cancer, diabetes, and obesity (12–15), which might be exacerbated by a sedentary lifestyle and physical inactivity, the WHO reiterated the importance of PA and provided specific guidelines during the pandemic (16). On a psychological level, PA was considered a key strategy to cope with distress associated with the implementation of preventive measures such as lockdowns and quarantines (17, 18). Indeed, physical inactivity can also contribute to mental health conditions that in turn are known to further encourage sedentary behaviours (19), such as depressive symptoms.

Nevertheless, several studies showed that during the COVID-19 pandemic mental health was deeply affected (20, 21), and PA levels substantially decreased while sedentary behaviours increased in several countries (22–24), also in younger age groups (25). Even prior to the COVID-19 pandemic, physical inactivity was recognised as a global health concern (26), with younger individuals also being affected (27). Since this might entail detrimental consequences on the overall well-being of individuals, and risks associated with physical inactivity might as well affect public health in the long term, it is crucial to address this issue across all ages and countries.

Italy was the first country outside of China where SARS-CoV-2 local transmission was detected and that soon implemented some of the strictest anti-COVID-19 measures (28, 29). However, our previous study (30) showed that there is no evidence that shutdown and limitation of sports activities were effective in reducing the spread of COVID-19 in Italy in young Italian athletes, namely children, adolescents, and young adults. Additionally, our findings indicated an increase in Body Mass Index (BMI) within this demographic. In order to deepen these results, we further investigated the impact of the interruption of sports activities on mental health, which is the focus of the present paper. The primary hypothesis asserted that levels of PA performed during the beginning of the second European and North American pandemic wave and the following months are associated with a different mental health status among young Italian non-professional athletes. Secondary aims regard the evaluation of possible differences concerning mental health with respect to sociodemographic variables (such as gender and educational level) and screen time.

## Materials and methods

### Procedures and sampling

Between June and September 2021, a national retrospective survey-based cross-sectional study involving children (≥6 years), adolescents, and young adults (aged ≤ 25 years) who used to play sports before the COVID-19 pandemic at a non-professional level was conducted. Data were collected via Google Forms and the link to the questionnaire was sent to Italian sports clubs and centres and shared on social media platforms. The survey administration was preceded by a 2-week pilot phase in which the reliability of the questionnaire and the clarity of the questions were assessed.

For participants under the age of 13, completion of the questionnaire required the involvement of legal guardians, while participants aged 13 and above were able to independently complete the questionnaire.

#### Eligibility criteria

In order to be included in the study, participants needed to meet specific inclusion criteria. These criteria included: (a) being aged between 6 and 25 years old, (b) not being professional athletes, and (c) providing informed consent. Participants who were 18 years or older were required to provide their own informed consent, while legal guardians were responsible for providing informed consent for participants under the age of 18. Conformity with these criteria was assessed through specific questions at the beginning of the questionnaire. As a result, participants who met the inclusion criteria were able to fully complete the questionnaire. Instead, individuals who were younger than 6 or older than 25 years, professional athletes, or those who did not provide informed consent were not permitted to proceed with the questionnaire.

#### Sample selection

Among the initial sample of 2,910 individuals who intended to complete the questionnaire, a total of 790 participants were unable to proceed. This was due to either their failure to provide the required informed consent (*n* = 5) or their non-conformity with the inclusion criteria (*n* = 540 participants older than 25 years; *n* = 245 professional athletes). After conducting quality checks to evaluate the completeness and validity of responses, 356 submissions were excluded due to incomplete/unrealistic answers. From the remaining 1,764 submissions, 47 were excluded due to missing responses for the mental health assessment. Consequently, a total of 1,717 submissions were used for the statistical analyses presented in this paper (Supplementary Figure 1).

#### Sample representativeness

The collected sample has proven to be representative of young Italian athletes to a good extent. In fact, according to a report released by the Italian National Olympic Committee (CONI) (31), the geographical representativeness of our sample closely aligns with the national distribution in 2020. Specifically, the CONI observed that 56% of athletes were situated in northern Italy, while 22% were located in central Italy, and another 22% in southern Italy/islands. In our study, 64.5% of the sample was located in northern Italy, 16.1% in the central Italy, and 15.5% in southern Italy/islands (see Table 1). A good representativeness was also achieved in terms of age. Indeed, national data concerning the age distribution of young Italian athletes (<35 years) indicates that young adults (age range: 18–35 years) constitute roughly one-third of the total, mirroring the findings of our study. Specifically, in our study, youth/young adults (age range: 16–25 years) accounted for 24.8% (*n* = 1,292) of the overall sample, while children/adolescents (age range: 6–15 years) constituted 75.2% (*n* = 425). Regarding gender, Italian data report a higher percentage of male athletes (71.8%), whereas our study shows a good balance between both genders (53.9% males and 44.6% females). Notably, the CONI sample includes professional athletes, a category that was excluded from the present study. Nevertheless, it is important to highlight that data from both the CONI and our study refer to the pandemic period.

**Table 1:** Characteristics of overall participants and by number of weekly training sessions.

		**Weekly training sessions**			
	**Overall (*****n*** = **1,717)**	**0 (*****n*** = **479)**	**1–2 (*****n*** = **834)**	>**2 (*****n*** = **404)**	***p*** **-value**
**Gender**					**< 0.001**
Male	926 (53.9)	218 (45.5)	503 (60.3)	205 (50.7)	
Female	766 (44.6)	252 (52.6)	325 (39.0)	189 (46.8)	
Missing	25 (1.5)	9 (1.9)	6 (0.7)	10 (2.5)	
**Compliance with WHO guidelines for PA**					**< 0.001**
Yes	568 (33.1)	112 (23.4)	228 (27.3)	228 (56.4)	
No	1,138 (66.3)	362 (75.6)	602 (72.2)	174 (43.1)	
Missing	11 (0.6)	5 (1.0)	4 (0.5)	2 (0.5)	
**Outdoor PA**					**< 0.001**
Yes	1,173 (68.3)	212 (44.3)	652 (78.2)	309 (76.5)	
No	538 (31.3)	265 (55.3)	179 (21.5)	94 (23.3)	
Missing	6 (0.4)	2 (0.4)	3 (0.3)	1 (0.2)	
**Sport**					**< 0.001**
Contact sports	685 (39.9)	126 (26.3)	415 (49.8)	144 (35.6)	
Non-contact sports	966 (56.3)	292 (61.0)	415 (49.8)	259 (64.1)	
Missing	66 (3.8)	61 (12.7)	4 (0.4)	1 (0.2)	
**Educational level**					**< 0.001**
Kindergarten, primary school	679 (39.5)	178 (37.2)	417 (50.0)	84 (20.8)	
Middle school	425 (24.8)	93 (19.4)	206 (24.7)	126 (31.2)	
Technical or professional institute	76 (4.4)	13 (2.7)	29 (3.5)	34 (8.4)	
High school	298 (17.4)	74 (15.4)	112 (13.4)	112 (27.7)	
University	152 (8.9)	87 (18.2)	32 (3.8)	33 (8.2)	
Currently not attending school	74 (4.3)	34 (7.1)	26 (3.1)	14 (3.5)	
Missing	13 (0.7)	0 (0)	12 (0.2)	1 (0.2)	
**Geographical area**					**< 0.001**
Southern Italy/Islands	266 (15.5)	115 (24.0)	98 (11.8)	53 (13.1)	
Central Italy	276 (16.1)	52 (10.9)	122 (14.6)	102 (25.2)	
Northern Italy	1,107 (64.5)	293 (61.2)	585 (70.1)	229 (56.7)	
Abroad	30 (1.7)	7 (1.5)	14 (1.7)	9 (2.2)	
Missing	38 (2.2)	12 (2.5)	15 (1.8)	11 (2.7)	
**Outdoor spaces at home**					**< 0.001**
Yes	958 (55.8)	203 (42.4)	492 (59.0)	263 (65.1)	
No	743 (43.3)	271 (56.6)	332 (39.8)	140 (34.7)	
Missing	16 (0.9)	5 (1.0)	10 (1.2)	1 (0.2)	
**Screen time (hours)**					**< 0.001**
≤ 2 h	427 (24.9)	87 (18.2)	229 (27.4)	111 (27.5)	
2–4 h	398 (23.2)	114 (23.8)	209 (25.0)	75 (18.6)	
>4 h	662 (38.6)	209 (43.6)	284 (34.1)	169 (41.8)	
Missing	230 (13.3)	69 (14.4)	112 (13.5)	49 (12.1)	

Frequencies and percentages (in parentheses) are reported in the table. Significant values are in bold.

#### Ethics statement

The study was conducted in agreement with the national and international regulations and the Declaration of Helsinki (2000). Approval by the Ethics Committee was not required as the online survey was completely anonymous, and it was not possible to keep track of any identifiable personal data. Further details regarding the procedure can be found in the previous publication (30).

### Materials

The questionnaire was developed by a panel of experts with in-depth knowledge in different subjects, such as epidemiology, psychology, and nutrition. It was administered in Italian language and required ~12–15 min to be completed. The questionnaire included multiple choice and open-ended questions covering six different areas: (a) SARS-CoV-2 infections, (b) socio-demographic information, (c) sports practice and level of PA, (d) mental health, (e) diet, and (f) screen time. The original version of the questionnaire has been previously published (30). The present paper is focused on the results regarding the following areas and corresponding aspects.

*Sociodemographic information:* participants were asked to report their gender, age, geographical area, currently attended school year, presence of outdoor spaces where to exercise at home between September 2020 and May 2021.

*Sports practice*: practice of any sports activity between September 2020 and May 2021, sport type, weekly frequency of training sessions, participation in sports competitions and/or activities organised by sports societies and centres, individual/team training, indoor/outdoor PA.

*Level of PA* was evaluated by using an adaptation of the International Physical Activity Questionnaire (32) in its short form (IPAQ-SF), a widely used 7-item self-report measure of habitual PA, whose reliability and validity have been confirmed in different countries (33). In this context, time spent on PA was recorded according to three intensity levels (vigorous, moderate, and light), along with the time spent in a sedentary position, in the period of COVID-19 waves instead of the previous week. This questionnaire was used to define compliance to WHO guidelines (34) according to athletes' age.

*Mental health* was investigated through two questionnaires: the Strengths and Difficulties Questionnaire (SDQ) (35) and the Psychological General Well-Being Index—Short Version (PGWB-S) (36). The SDQ is a 25-item scale with response options provided on a 3-point Likert-type scale (from 0 to 2) widely used to screen children and adolescents' mental health in terms of psychological difficulties. This scale allows evaluating emotional symptoms, conduct problems, hyperactivity/inattention, peer relationship problems, and prosocial behaviour. The first four subscales can be added together to generate a total difficulties score, and higher scores indicate a higher level of symptomatology. As for the latter subscale, higher scores indicate higher prosocial behaviour. The parent version was used for participants up to the age of 12, whereas the self-report version was administered to participants aged 13 and older. In this study, Cronbach's alpha for both versions of the SDQ was 0.85, indicating good internal consistency. The PGWB-S is a 6-item health-related Quality of Life (HRQoL) questionnaire that investigates psychological general well-being in youth and adulthood and was therefore used to assess participants' mental health from 16 years of age. Response options are provided on a 6-point Likert-type scale (from 0 to 5), and higher scores indicate higher levels of psychological well-being. Cronbach's alpha for the PGWB-S in this study was 0.90, showing high internal consistency.

*Screen time*: participants were asked to report the amount of time spent on screens daily in terms of hours between September 2020 and May 2021, including television, personal computer, tablet, smartphone, and videogames.

The validated questionnaires used for the present study are summarised in Supplementary Table 1.

### Variables definition

In order to assess the association between PA/sports activity and mental health during the study period, we defined different variables. Specifically, two different variables were defined with respect to PA/sports activity: (1) *training during periods of openings/closures*, which refers to PA/sports activity carried out only in periods of openings (September–October 2020 and April–May 2021), also throughout the period of lockdowns and closures (November 2020–March 2021), or none; and (2) *weekly training sessions*, which refers to the average number of weekly trainings. Continuous training sessions refer to PA carried out both during periods of closures and during periods of reopening. Non-continuous training sessions refer to PA carried out only during periods of openings. Furthermore, compliance with the WHO guidelines measured with the IPAQ-SF was assigned the value of 1, and non-compliance was assigned the value of 0. Compliance or non-compliance were specifically assessed by considering duration and frequency of moderate and vigorous-intensity activities per week, as well as age, as reported (34).

As for mental health, all the scores for the SDQ (35) and for the PGWB-S (36) were considered both as continuous and categorical variables. When considered as continuous variables, as previously stated, higher scores for the SDQ indicate higher levels of difficulties/symptomatology, whereas higher scores for the PGWB-S indicate higher levels of psychological well-being. However, in order to clarify the treatment of scores as categorical variables, further explanations are required. Indeed, scores for children/adolescents were categorised into three groups (i.e., normal, borderline, and abnormal) following the 3-band categorisation from the SDQ scoring guidelines. Subsequently, borderline and abnormal scores were merged for the present analyses. The four subscales related to psychological difficulties (emotional symptoms, conduct problems, hyperactivity/inattention, peer relationship problems) were analysed both individually and combined to create a total difficulties score. Psychological difficulties for each subscale and for the total score were intended as scoring as borderline or abnormal. Conversely, prosocial behaviour was considered as absent when scoring as borderline or abnormal. For youth and young adults, the median for the PGWB-S was calculated (Median = 14) to separate the half of the sample showing higher levels of psychological well-being from the half that showed lower levels. Since the items of the PGWB-S evaluate psychological well-being by assessing the presence/absence of psychological symptoms, those who scored below the median were considered to be experiencing psychological symptoms. When presenting and discussing the overall results of the study, psychological difficulties measured for children/adolescents and levels of psychological well-being below the median measured for youth/young adults are collectively referred to as psychological symptoms.

Note that, in Italy, children who are 6 years old may attend either kindergarten or primary school. Consequently, we merged the data of children aged 6 who were attending kindergarten with those attending primary school.

### Statistical analyses

Separating the analyses based on the type of questionnaire used to assess mental health between (SDQ for children/adolescents aged 6–15 years, and PGWB-S for youth/young adults aged 16–25 years), multivariable logistic regression models were used to estimate the risk of psychological difficulties; odds ratios (ORs) with 95% confidence intervals (CIs) were calculated. Scores both for the SDQ and each subscale and for the PGWB-S were considered as continuous variables for the boxplots.

Besides gender, age range (i.e., educational level), and geographical area, significant variables identified during the univariate analysis were included as co-factors in the multivariable analysis.

Chi-square test, Kruskal-Wallis test, and Wilcoxon-sum rank test were performed to investigate the association between variables regarding training and the participants' characteristics depending on the nature of the variable.

*P*-values < 0.05 were considered statistically significant. The analyses were performed by using the statistical software R (version 4.1.1).

## Results

Characteristics of the subjects in terms of weekly training sessions are presented in Table 1. Females reported training 1–2 times/week significantly less compared to males (39.0% vs. 60.3%), while demonstrating similar levels of intensive weekly training sessions (>2 times/week). Furthermore, individuals training >2 times/week reported significantly lower screen time (27.5% ≤ 2 h) than those who did not train (18.2%). Similar results were obtained considering periods of openings and closures (Supplementary Table 2).

### Children and adolescents

**Figure 1:**
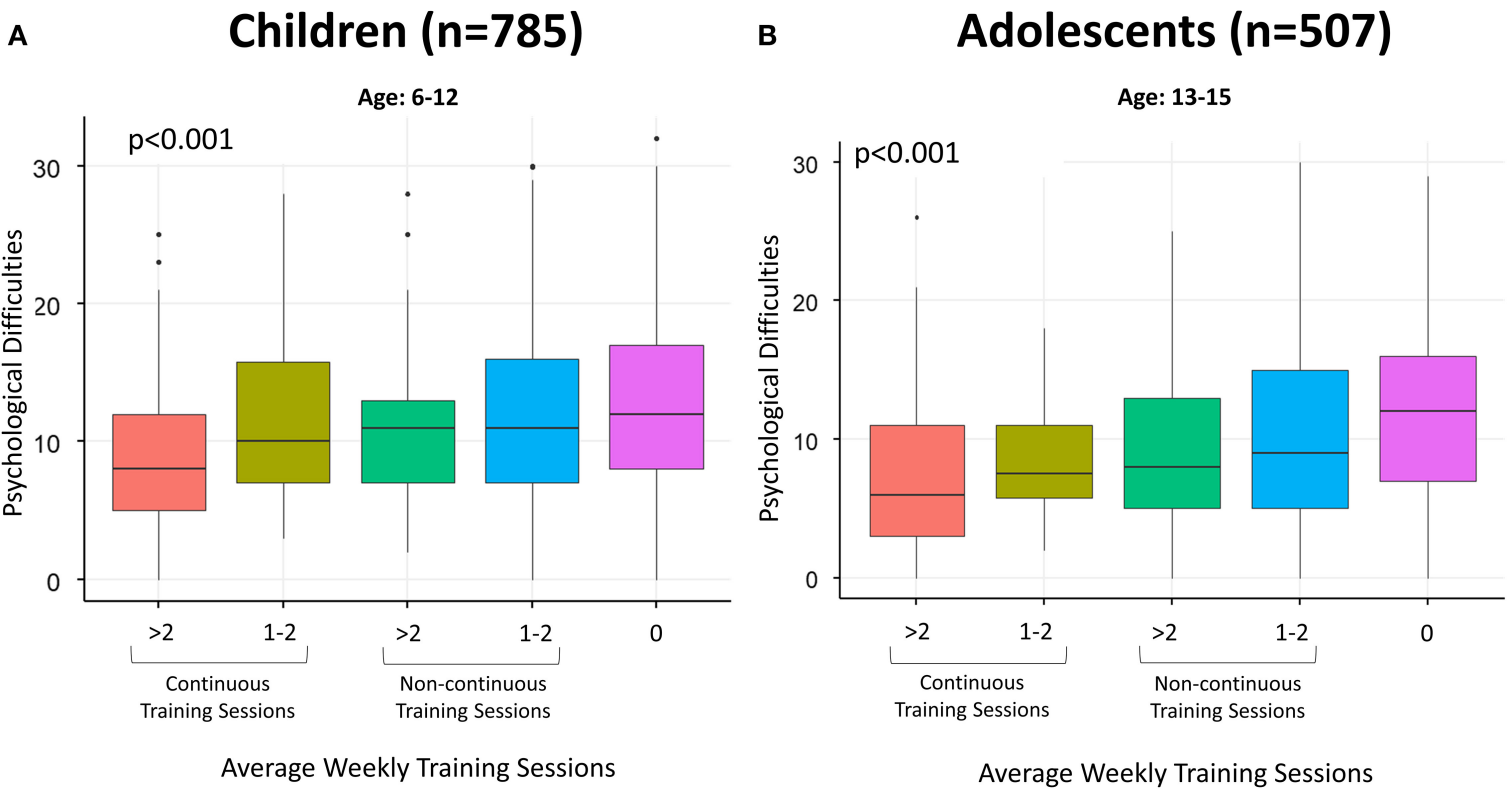
**(A, B)** Boxplots regarding psychological difficulties in terms of average weekly training sessions (continuous vs. non-continuous) in children **(A)** and adolescents **(B)**. Psychological difficulties were calculated by adding together continuous scores from the subscales referring to emotional symptoms, conduct problems, peer relationship problems, and hyperactivity/inattention of the SDQ (35). Higher scores indicate a higher level of symptomatology. Continuous training sessions refer to PA carried out both during periods of closures and during periods of openings. Non-continuous training sessions refer to PA carried out only during periods of openings.

**Figure 2:**
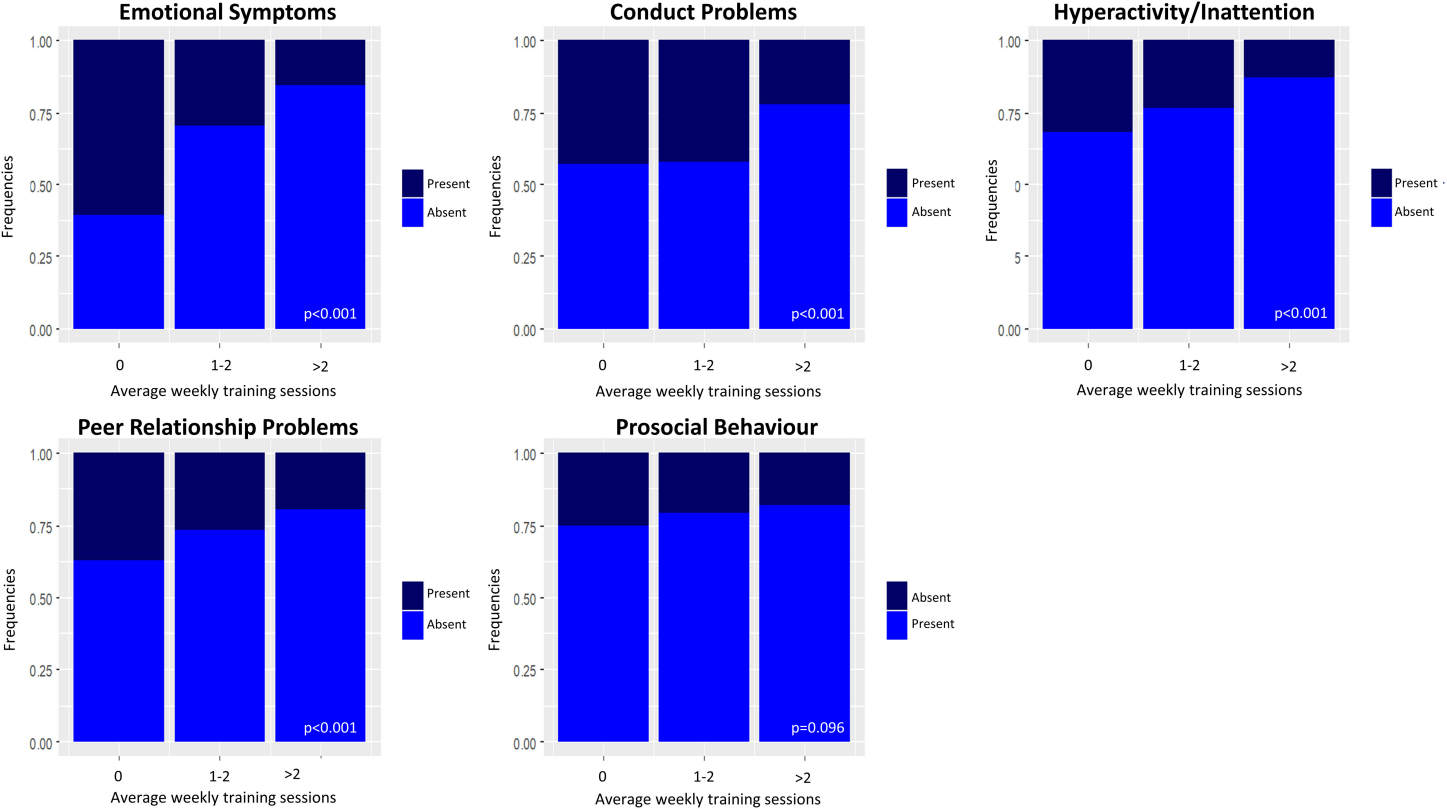
Relative frequencies of psychological difficulties in children and adolescents by average weekly training sessions. Presence/absence for the subscales referring to psychological difficulties (emotional symptoms, conduct problems, hyperactivity/inattention, and peer relationship problems) and prosocial behaviour were determined using the 3-band categorisation (37) suggested in the scoring guidelines for the Strengths and Difficulties Questionnaire (SDQ) (35). Absence for the four subscales related to psychological difficulties indicates a normal score, while presence indicates a borderline or abnormal score. Conversely, absence for the prosocial behaviour scale indicates a borderline or abnormal score, while presence indicates a normal score.

**Figure 3:**
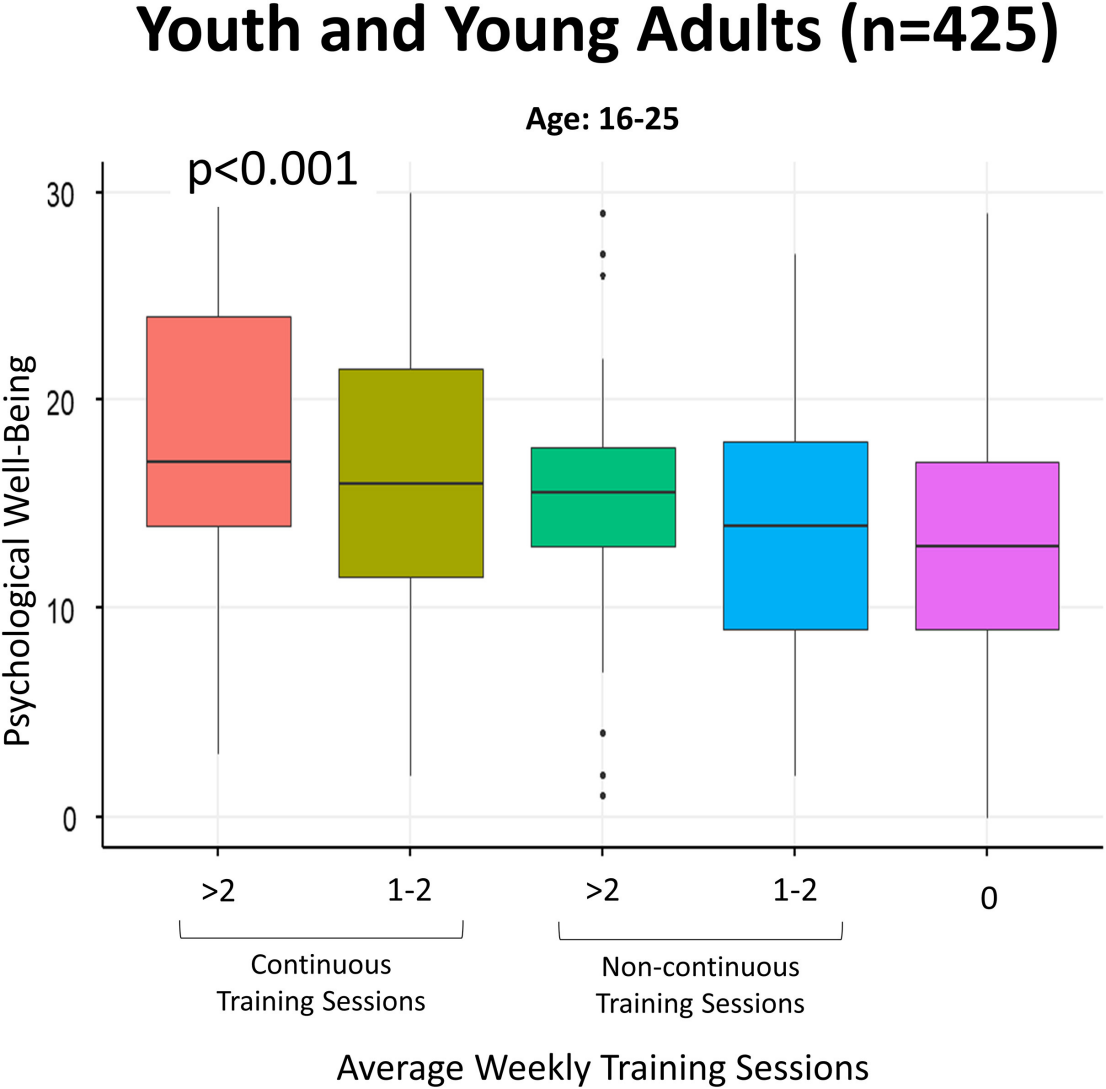
Boxplots regarding psychological well-being in terms of average weekly training sessions (continuous vs. non-continuous) in youth and young adults. Psychological well-being was calculated by using the continuous score obtained through the PGWB-S (36), and higher scores indicate a higher level of psychological well-being. Continuous training sessions refer to PA carried out both during periods of closures and during periods of reopening. Non-continuous training sessions refer to PA carried out only during periods of openings.

The association of sports activity and other covariables with mental health for children and adolescents and for youth and young adults are presented separately in Supplementary Tables 3, 4 and Figures 1–3, respectively. ORs and 95% CIs are presented indicating the strength of the association of sports activity with psychological difficulties and with each subscale of the SDQ, adjusted for possible confounding factors presented in Table 1 and Supplementary Table 2.

As shown in Figures 1A, B, training more than twice a week, especially in a continuous way, is associated with lower levels of psychological difficulties in children and adolescents. On the other hand, those who did not train during the study period showed higher levels of psychological difficulties. As shown in Supplementary Table 3, carrying out >2 training sessions per week compared to none is inversely associated with psychological difficulties (OR = 0.54, 95% CI 0.35–0.82) for children and adolescents. Being male, not adherent to WHO guidelines for PA, living in a house with no outdoor space and using electronic devices >2 h/day were found to be significantly associated with psychological difficulties, as well as not engaging in outdoor PA. Psychological difficulties were more prevalent in kindergarten and primary school students than middle and high school students.

Results regarding each subscale of the SDQ are presented in Table 2.

**Table 2:** Estimates of association from multivariable model regarding each subscale of the SDQ in children and adolescents.

	**Emotional symptoms**	**Conduct problems**	**Hyperactivity/ inattention**	**Peer relationships problems**	**Lack of prosocial behaviour**
**Variables**	**OR (95% CI)**	**OR (95% CI)**	**OR (95% CI)**	**OR (95% CI)**	**OR (95% CI)**
**Average weekly trainings (days)**					
>2 vs. 0	**0.54 (0.36–0.81)** ^**^	**0.63 (0.43–0.92)** ^*^	**0.61 (0.44–0.84)** ^**^	-	-
1–2 vs. 0	**0.72 (0.54–0.97)** ^*^	1.17 (0.87–1.58)	0.73 (0.49–1.07)	-	-
**Compliance with WHO guidelines for PA**					
Yes vs. no	**0.61 (0.44–0.83)** ^**^	-	**0.56 (0.39–0.78)** ^***^	**0.53 (0.38–0.73)** ^***^	**0.72 (0.52–1.00)** ^*^
**Outdoor PA**					
Yes vs. no	-	**0.66 (0.50–0.87)** ^**^	**0.68 (0.52–0.90)** ^***^	**0.58 (0.45–0.76)** ^***^	**0.70 (0.52–0.94)** ^*^
**Gender**					
Males vs. females	0.93 (0.73–1.21)	**1.45 (1.14–1.86)** ^**^	**1.82 (1.38–2.43)** ^***^	1.13 (0.87–1.46)	**1.51 (1.14–2.01)** ^**^
**Education**					
Technical or professional institute vs. High school	0.97 (0.21–3.20)	1.61 (0.61–3.99)	1.64 (0.43–5.16)	0.60 (0.16–1.73)	2.19 (0.91–5.13)
Kindergarten, primary school vs. High school	**5.49 (3.23–9.79)** ^***^	**3.59 (2.32–5.69)** ^***^	**3.98 (2.26–7.61)** ^***^	**2.07 (1.32–3.32)** ^**^	1.01 (0.65–1.60)
Middle school vs. High school	**2.77 (1.62–4.98)** ^***^	**1.64 (1.04–2.64)** ^*^	**2.03 (1.11–3.98)** ^*^	**1.67 (1.05–2.70)** ^*^	0.84 (0.52–1.37)
**Geographical area**					
Abroad vs. Northern Italy	1.83 (0.49–6.56)	0.86 (0.21–3.03)	3.18 (0.85–11.4)	1.46 (0.37–5.09)	1.61 (0.34–5.78)
Central vs. Northern Italy	0.94 (0.65–1.34)	1.12 (0.80–1.56)	0.88 (0.59–1.30)	0.92 (0.64–1.31)	1.01 (0.68–1.46)
Southern Italy Islands vs. Northern Italy	1.17 (0.82–1.67)	**1.86 (1.32–2.62)** ^***^	1.07(0.73–1.57)	**1.43 (1.01–2.01)** ^*^	1.31 (0.89–1.89)
**Outdoor spaces at home**					
Yes vs. no	**0.64 (0.50–0.82)** ^**^	**0.66 (0.52–0.84)** ^***^	-	-	-
**Screen time (hours)**					
>4 vs. ≤ 2 h	**1.92 (1.37–2.71)** ^***^	-	-	**2.00 (1.41–2.84)** ^***^	-
2–4 h vs. ≤ 2 h	**1.44 (1.03–2.02)** ^*^			**1.77 (1.25–2.52)** ^**^	

OR, Odds Ratio; CI, Confidence Interval.

Odds Ratios (ORs) and 95% Confidence Intervals (CIs) for significant variables for each subscale were reported. All the models were adjusted by gender, age range (i.e., educational level), and geographical area. The event consisted of presenting the problem corresponding to each subscale, i.e., scoring as borderline or abnormal according to the 3-band categorisation suggested in the scoring guidelines (37) for the SDQ (35).

* p ≤ 0.05,

** p ≤ 0.01,

*** p ≤ 0.001. Significant values in bold.

Figure 2 shows that children and adolescents who have not exercised show more frequent emotional symptoms, conduct problems, hyperactivity/inattention, and peer relationships problems compared to those who have exercised. The difference is particularly noticeable when comparing those who did not exercise and those who exercised more than twice a week.

#### Emotional symptoms

Exercising is significantly inversely associated with emotional symptoms (1–2 times/week vs. 0: OR = 0.72, 95% CI 0.54–0.97; >2 times/week vs. 0: OR = 0.54, 95% CI 0.36–0.81), along with having outdoor spaces at home. Spending >2 h/day using electronic devices is significantly directly associated with emotional symptoms, which are more prevalent in kindergarten/primary school and middle school compared to high school. No association emerged with gender and geographical area.

#### Conduct problems

Variables significantly inversely associated with conduct problems are the following: exercising >2 times/week (OR = 0.63, 95% CI 0.43–0.92), outdoors PA during the period between September 2020 and May 2021, and having outdoor space at home. Males showed more conduct problems than females. A difference emerged in terms of conduct problems between education levels: kindergarten, primary school and middle school students show more conduct problems than high school students. Finally, children and adolescents from Southern Italy/Islands reported more conduct problems than North Italians.

#### Hyperactivity/inattention

Exercising more than twice a week (OR = 0.61, 95% CI 0.44–0.84), having carried out at least 1 training session during the period of closures (OR = 0.61, 95% CI 0.44–0.84; result not shown), outdoor PA, and being physically active according to WHO guidelines are significantly inversely associated with hyperactivity/inattention. Males compared to females are more prone to hyperactivity/inattention. A difference in terms of hyperactivity/inattention also emerged among educational levels: subjects in kindergarten/primary school and middle school are at higher risk of presenting hyperactivity/inattention than students in high school.

#### Peer relationship problems

Being physically active according to WHO guidelines and engaging in outdoor PA during the study period are inversely associated with peer relationship problems. Those living in Southern Italy/Islands and those using electronic devices for >2 h/day are more prone to peer relationship problems. A difference in terms of peer relationship problems emerged between educational levels, with higher problems reported for children compared to adolescents, while no significant differences emerged for gender.

#### Prosocial behaviour

Outdoor PA and compliance to WHO guidelines are associated with lower probability of lack of prosocial behaviour. As for gender, males are less likely to show prosocial behaviour than females. No association emerged with educational levels and geographical area.

### Youth and young adults

Results regarding youth and young adults are reported in Supplementary Table 4. Training >2 times/week vs. zero is inversely associated with psychological symptoms (OR = 0.25, 95% CI 0.14–0.45), as well as training during the period of closures (OR = 0.35, 95% CI 0.21–0.57, results not shown) and being male. Using electronic devices >2 h/day is significantly associated with psychological symptoms.

Figure 3 shows that exercising twice a week is associated with higher levels of psychological well-being, especially compared to those who have not exercised, in participants aged 16–25 years.

## Discussion

The current study sought to investigate the relationship between PA and psychological health in various young age groups during the COVID-19 pandemic. Particularly, levels of PA performed during the beginning of the second European and North American pandemic wave and the following months were expected to be associated with a different mental health status among young Italian non-professional athletes. Our findings confirmed this hypothesis, revealing that not engaging in PA was associated with a higher incidence of psychological symptoms in both children/adolescents and youth/young adults. Those who were able to train continuously, both during closures/lockdowns and openings, showed fewer psychological symptoms.

In addition, engaging in outdoor PA or having access to outdoor spaces at home appears to be particularly crucial for children and adolescents. This is in line with previous studies highlighting an association between outdoor activities and psychological well-being (38–42). As suggested by previous research (43, 44), these findings emphasise the need to facilitate outdoor recreation opportunities, which should be taken into account for younger age groups during times of crisis in Italy as well. It should also be considered that outdoor activities reduce the probability of transmission of respiratory viruses (45, 46).

Furthermore, screen time has been found to be associated with psychological symptoms in children and adolescents, as well as youth and young adults. As we discovered, excessive use of electronic devices has a negative impact on psychological well-being (47–50) and is associated with a decrease in PA (51, 52). Over 2 h of daily screen time, in particular, was linked to emotional and peer relationship problems in children/adolescents. Indeed, studies show that increased screen time is linked to lower levels of social competence (53, 54), as well as higher levels of depression and anxiety symptoms (55–57). It is to note that emotional and social competences and PA/sports show a reciprocal relationship, with one positively influencing the other. In fact, PA and sports can enhance emotional and social competences (58, 59), explaining why exercise resulted as linked to the adoption of prosocial behaviour in children/adolescents as well. It is to note that possessing high levels of these competences (such as self-control, empathy, and communication) can in turn improve one's likelihood of engaging in PA and sports activities. Social contact and support from others due to sports activities increases the beneficial effects of physical exercise interventions (60), and these benefits should be taken into account (17). In fact, in a Norwegian cohort study involving 382 children, it was found that there was no clear link between post COVID-19 conditions and the previous infection. Instead, loneliness and lack of PA were shown to be important factors. However, it is important to note that the increase in remote learning has contributed to the amount of screen time.

The current study also discovered significant gender differences. Indeed, male children and adolescents were more prone to present psychological difficulties overall—specifically, conduct problems, hyperactivity/inattention, and lower prosocial behaviour. This is consistent with research showing that males exhibit externalising problems more frequently than females (i.e., dysfunctional acting-out and outward behaviours related to poor impulse control, such as rule-breaking, aggression, and impulsivity) (61–65). However, young adult males presented less psychological symptoms than young adult females. This is in line with previous findings (66) and might be due to the fact that females reported training less than males during the study period.

In a study conducted on 3,245 children and adolescents (67), caregivers reported behavioural changes in 64.3% of children under 6 years old and in 72.5% of those between the ages of 6 and 18 during the COVID-19 pandemic. In both age groups, distress linked to quarantine was significantly associated with such behavioural changes. However, in our study more psychological difficulties were found among children in kindergarten and primary school students, along with middle school students, compared to high school. Indeed, children attending kindergarten, primary and middle schools were more frequently reported as not having trained at all during the study period.

Along with the positive effects of PA on mental health, its beneficial outcomes on physical health, specifically concerning infectious diseases, should be considered as well. By boosting the immune system, PA can help minimise the adverse effects of the infectious process caused by COVID-19 (68). Recent publications showed a reduction in aggravation of the disease, in hospital admissions and a decrease of death from COVID-19 (69, 70). Furthermore, PA can also prevent COVID-19 infection, and the mechanisms can be partially explained by the higher concentration of immune cells, such as T lymphocytes, and the increased resistance of the mucosal immune barrier (salivary IgA immunoglobulin), observed in more physically active individuals (71, 72), providing a greater immunity against different types of viruses and bacteria entering the human body through the oral cavity and upper airways.

Fortunately, COVID-19 showed a very good prognosis in youth (73), and young index cases were found to be significantly less likely than adults to favour viral spread (74). In fact, it has been shown that the increased exposure to various types of viruses during childhood, including some cold coronaviruses, increased the immune response to SARS-CoV-2 (75).

All these factors should be duly considered when determining the appropriate preventive measures to be implemented for the younger demographic, given the impact on the psychological dimensions, which could affect the adoption of health behaviours.

A limitation of this study is that data were collected retroactively by using a self-administered web survey, raising questions about the accuracy of such information. However, the high interpretability of our results and the agreement with previous research suggest that the overall standard was high. Although other unmeasured confounders might affect the outcomes, key athletes' and sport-related characteristics were taken into account, as well as several sociodemographic variables, and a longer questionnaire might have led to lower compliance and accuracy. Lastly, the findings reported in the present paper refer to the young Italian population and may not necessarily apply to other regions and age groups. However, our results are in line with previous studies conducted in other countries investigating the impact of PA on psychological health during the COVID-19 pandemic (43, 76–80).

Ultimately, the present study confirms the positive impact of PA on mental health during the COVID-19 pandemic among younger age groups, and limitation of sports activities as a preventive measure should be considered in light of the associated risks. Indeed, these findings also provide further understanding of the risk-benefit relationship of interrupting sports activities as a preventive measure against contagion, having significant implications for policymakers and healthcare professionals in developing effective strategies to promote PA and mental health in younger populations. Particularly, the psychosocial aspects most related to PA identified in this study can be helpful to identify alternative and targeted solutions for future epidemics/pandemics. Although these results can provide valuable insights on possible solutions, further research is needed to develop effective interventions. The assessment of PA benefits and its promotion is particularly relevant in childhood and adolescents, as it is a critical period for the establishment of future lifestyles and the development of habits that exert a profound impact on overall health throughout the lifespan (81), also affecting public health in the long term.

## Conclusions

The current study provides additional evidence supporting the positive impact of PA on mental health among younger age groups during the COVID-19 pandemic. Moreover, it emphasises the importance of considering the potential risks associated with the restriction of sports activities as a preventive measure. Specifically, by assessing the psychosocial dimensions closely linked to PA, targeted strategies can be developed. These findings contribute to a better understanding of the global situation and can be valuable in enhancing the management of infectious diseases in the future.

## Data availability statement

The raw data supporting the conclusions of this article will be made available by the authors, without undue reservation.

## Ethics statement

The study was conducted in agreement with the national and international regulations and the Declaration of Helsinki (2000). Approval by the Ethics Committee was not required as the online survey was completely anonymous, and it was not possible to keep track of any identifiable personal data. The studies were conducted in accordance with the local legislation and institutional requirements. Written informed consent for participation in this study was provided by the participants' legal guardians/next of kin. No potentially identifiable images or data are presented in this study.

## The EuCARE WP4

We want to mention the members of the EuCARE WP4, who supported the development of this study, which is part of the EuCARE Project: Francesca Incardona (1), Chiara Mommo (1), Gibran Horemheb Rubio Quintanares (2, 3, 4), Ana Abecasis (5), Daniela Alves (5), Inês Cruz Alves (5), Marta Pingarilho (5) Pier Luigi Lopalco (6), Susanna Chiocca (7), Ilaria Cutica (8), Davide Mazzoni (8), Nuno Amparo (5), Daniela Carmagnola (9), Claudia Dellavia (9), Gianvincenzo Zuccotti (10, 11), Felix Dewald (2), Rolf Kaiser (2), Nils Bardeck (2), Michael Böhm (2), Michal Rosen-Zvi (12), Yishai Shimoni (12), Sivan Ravid (12), Tal Kozlovski (12), Sofia Seabra (5), Victor Pimentel (5), Mafalda Miranda (5), Giuseppina Tucci (13), Carmen Romero (13), Francesco Vairo (14), Martina Spaziante (14), Valeria Gabellone (6), Giulia Vaglio (6), Fabrizio Fedele (6), Osvaldo Rafael Ramírez Ibarra (15), Victor Martín Escalante Gómez (15), and Mariela González Rodríguez (15).

(1) EuResist Network, Rome, Italy; (2) Institute of Virology, University Clinics of Cologne, Cologne, Germany; (3) Paul Ehrlich Institut, Langen, Germany; (4) Infectious Diseases Department, Instituto Nacional de Ciencias Médicas y Nutrición Salvador Zubitsn, Mexico City, Mexico; (5) Global Health and Tropical Medicine, GHTM, Associate Laboratory in Translation and Innovation Towards Global Health, LA-REAL, Instituto de Higiene e Medicina Tropical, IHMT, Universidade NOVA de Lisboa, UNL, Portugal; (6) Department of Biological and Environmental Sciences and Technology, University of Salento, Lecce, Italy; (7) Department of Experimental Oncology, IEO, European Institute of Oncology IRCCS, Milan, Italy; (8) Department of Oncology and Hemato-Oncology, University of Milan, Milan, Italy; (9) Department of Biomedical, Surgical and Dental Sciences, University of Milan, Milan, Italy; (10) Paediatric Department, Vittore Buzzi Children's Hospital, Milan, Italy; (11) Department of Biomedical and Clinical Science, University of Milan, Milan, Italy; (12) IBM Research, Mount Carmel Haifa, Israel; (13) Organising Bureau of European School Student Unions (OBESSU); (14) National Institute for Infectious Diseases, “Lazzaro Spallanzani” IRCCS, Rome, Italy; (15) Laboratorio Universitario de Diagnóstico Molecular, Universidad de Guanajuato, Mexico.

## Author contributions

ET, SR, and OD'E: conception, design, analysis and interpretation of data, and drafting and revising manuscript critically for important intellectual content. GT: conception, design, and drafting and revising manuscript critically for important intellectual content. FB and GC: analysis and interpretation of data and revising manuscript critically for important intellectual content. FG, PG, and SG: conception, design, interpretation of data, and revising manuscript critically for important intellectual content. MI, DR, AS, CS, GP, and NM: conception, design, and revising manuscript critically for important intellectual content. All authors provided final approval of the version to be published and agree to be accountable for all aspects of the work in ensuring that questions related to the accuracy or integrity of any part of the work are appropriately investigated and resolved.
