# Supplementary material for: Sports activity limitation during the COVID-19 pandemic in young Italian athletes: impact on mental health in children, adolescents, and young adults
Source: Front Public Health. 2023 Aug 10;11:1237443. doi: 10.3389/fpubh.2023.1237443 (PMC10448519; doi:10.3389/fpubh.2023.1237443)
Supplement: Supplementary file 1 [file Table_1.docx]

**SUPPLEMENTARY MATERIALS**

**Figure 1S. Flow chart illustrating the selection of the final sample.**

**
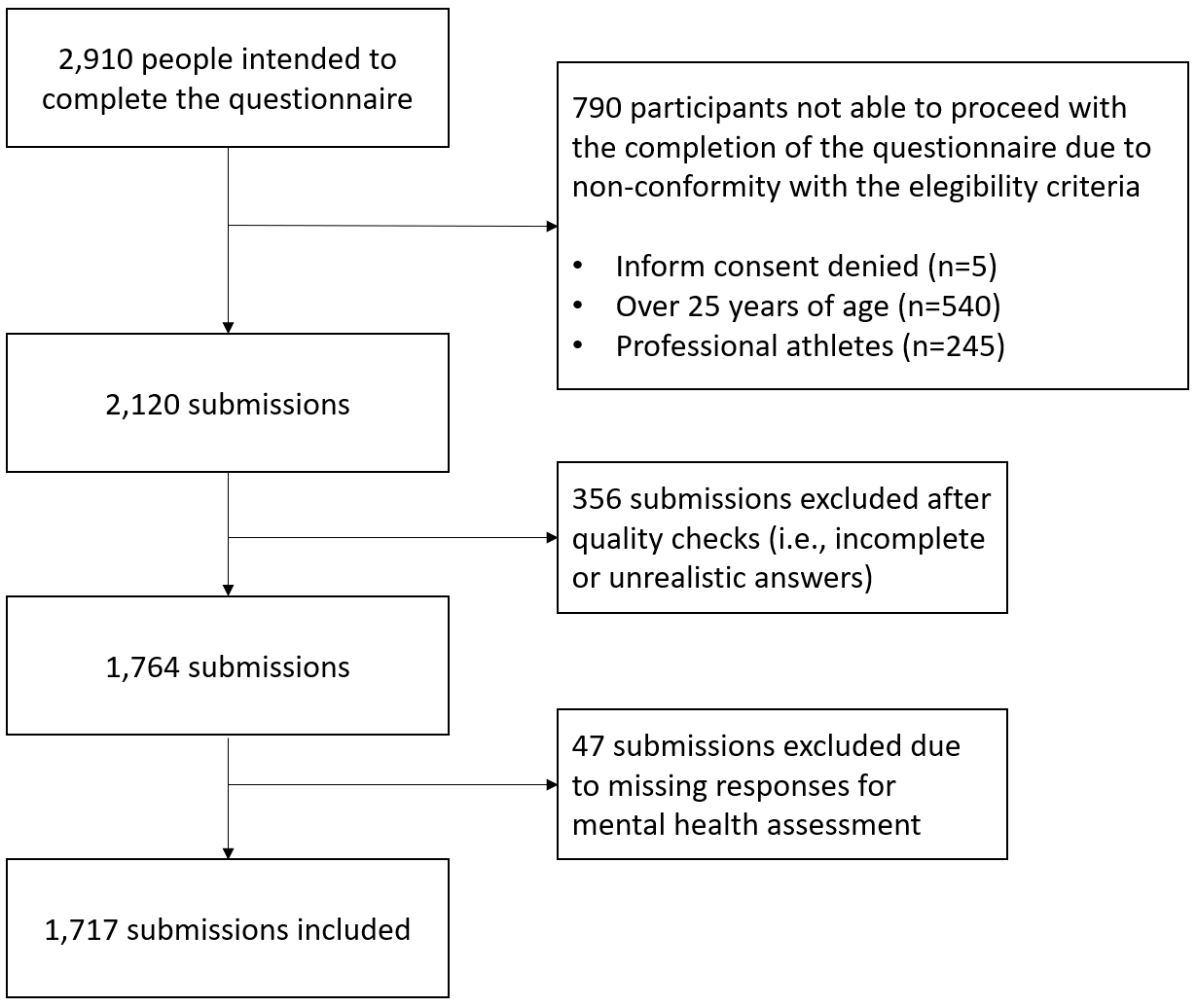
**

**Tab. 1S. Summary showing the validated scales used in the present study.**

| **Variable** | **Scale** | **Target (age range)** |
| --- | --- | --- |
| **Mental health** | SDQ – Parent version | Children (6-12) |
|  | SDQ – Self-report version | Adolescents (12-15) |
|  | PGWB-S | Youth/Young adults (16-25) |
| **Level of PA** | IPAQ | Whole sample |

Note. Sociodemographic characteristics, sports practice, and screen time were assessed
through *ad hoc* questions.

**Table 2S. Characteristics of individuals by training by periods of openings and closures.**

|  | **Training by periods of openings and closures** | | |  |
| --- | --- | --- | --- | --- |
|  | **None**  **(n=496)** | **At least 1 training during periods of openings (n=307)** | **At least 1 training during period of closures**  **(n=992)** | ***p*-value** |
| **Gender**  Male  Female  Missing | 224 (45.2)  263 (53.0)  9 (1.8) | 186 (60.6)  118 (38.4)  3 (1) | 549 (55.3)  429 (43.2)  14 (1.5) | **<0.001** |
| **Educational level**  Kindergarten, primary school  Middle school  Technical or professional institute  High school  University  Currently not attending school  Missing | 194 (39.1)  94 (18.9)  14 (2.3)  76 (15.3)  87 (17.5)  31 (6.9)  0 (0) | 174 (56.7)  70 (22.8)  12 (3.9)  34 (11.1)  10 (3.2)  7 (2.3)  0 (0) | 383 (38.6)  277 (27.9)  56 (5.6)  193 (19.4)  55 (5.4)  25 (2.5)  3 (0.6) | **<0.001** |
| **Compliance with WHO guidelines for PA**  Yes  No  Missing | 113 (22.8)  366 (73.4)  17 (3.8) | 76 (24.7)  225 (73.3)  6 (2) | 389 (39.3)  585 (59.0)  18 (1.7) | **<0.001** |
| **Geographical area**  South and Islands  Centre  North  Foreign or other  Missing | 117 (23.6)  55 (11.1)  304 (61.3)  7 (1.4)  13 (2.6) | 44 (14.3)  36 (11.7)  214 (69.7)  7 (2.3)  6 (2) | 115 (11.6)  193 (19.4)  643 (64.8)  16 (1.6)  25 (2.6) | **<0.001** |
| **Outdoor PA**  Yes  No  Missing | 220 (44.3)  273 (55.0)  3 (0.7) | 205 (66.8)  99 (32.2)  3 (1) | 804 (81.0)  187 (18.8)  1 (0.2) | **<0.001** |
| **Screen time (hours)**  ≤2h  2h-4h  >4h  Missing | 92 (18.5)  115 (23.2)  209 (42.1)  80 (16.2) | 85 (27.7)  75 (24.4)  114 (37.1)  33 (10.8) | 275 (27.7)  222 (22.3)  351 (35.4)  144 (14.6) | **0.002** |
| **Type of sport**  Contact sports  Non-contact sports  Missing | 136 (27.4)  294 (59.3)  66 (13.3) | 150 (48.8)  156 (50.8)  1 (0.4) | 444 (44.7)  544 (54.8)  4 (0.5) | **<0.001** |
| **Outdoor spaces at home**  Yes  No  Missing | 214 (43.1)  277 (55.8)  5 (1.1) | 174 (56.7)  131 (42.7)  2 (0.6) | 610 (61.5)  372 (37.5)  10 (1.0) | **<0.001** |

Frequencies and percentages (in parentheses) are reported in the table. Significant values are in bold.

**Table 3S. Factors associated with psychological difficulties* in children and adolescents and average weekly training sessions.**

| **Variables** | **OR^**^** | **95% CI** | **p-value** |
| --- | --- | --- | --- |
| **Average weekly training sessions (days)** |  |  |  |
| ≤2 vs 0 | 0.89 | (0.66, 1.22) | 0.5 |
| >2 vs 0 | 0.54 | (0.35, 0.82) | **0.004** |
| **Compliance with WHO guidelines for PA** |  |  |  |
| No vs Yes | 1.49 | (1.09, 2.07) | **0.015** |
| **Outdoor PA** |  |  |  |
| No vs Yes | 1.46 | (1.09, 1.95) | **0.011** |
| **Gender** |  |  |  |
| Males vs females | 1.37 | (1.06, 1.79) | **0.018** |
| **Education** |  |  |  |
| Middle school vs Kindergarten, primary school | 0.42 | (0.31, 0.57) | **<0.001** |
| High school vs Kindergarten, primary school | 0.35 | (0.21, 0.56) | **<0.001** |
| Technical or professional institute vs Kindergarten, primary school | 0.35 | (0.11, 0.89) | **0.041** |
| Currently not attending school vs Kindergarten, primary school | 0.62 | (0.13, 2.17) | 0.5 |
| **Outdoor spaces at home** |  |  |  |
| No vs Yes | 1.45 | (1.12, 1.88) | **0.005** |
| **Screen time (hours)** |  |  |  |
| 2h-4h vs ≤2 h | 1.82 | (1.28, 2.59) | **<0.001** |
| >4h vs ≤2 h | 2.41 | (1.69, 3.44) | **<0.001** |
|  |  |  |  |

Significant values in bold. OR= Odds Ratio; CI= Confidence Interval

* Psychological difficulties were calculated by adding together scores from the subscales referring to emotional symptoms, conduct problems, peer relationship problems, and hyperactivity/inattention. The event consisted of presenting psychological difficulties, i.e., scoring as borderline or abnormal according to the 3-band categorisation suggested in the scoring guidelines (37) for the SDQ (35).

**Multivariable analysis.

**Table 4S. Factors associated with psychological symptoms* in youth and young adults and weekly training sessions.**

| **Variables** | **OR^**^** | **95% CI** | ***p*-value** |
| --- | --- | --- | --- |
| **Average weekly training sessions (days)** |  |  |  |
| ≤ 2 vs 0 | 0.60 | (0.35, 1.01) | 0.055 |
| >2 vs 0 | 0.25 | (0.14, 0.45) | **<0.001** |
| **Compliance with WHO guidelines for PA** |  |  |  |
| No vs Yes | 1.05 | (0.66, 1.68) | 0.8 |
| **Gender** |  |  |  |
| Males vs females | 0.35 | (0.22, 0.55) | **<0.001** |
|  |  |  |  |
| **Education** |  |  |  |
| Technical or professional institute vs High school | 0.57 | (0.26, 1.20) | 0.14 |
| University vs High school | 0.83 | (0.48, 1.42) | 0.5 |
| Currently not attending school vs High school | 0.59 | (0.30, 1.18) | 0.14 |
| **Screen time (hours)** |  |  |  |
| 2h-4h vs ≤2 h | 5.73 | (2.33, 15.2) | **<0.001** |
| >4h vs ≤2 h | 6.89 | (3.15, 16.6) | **<0.001** |
|  |  |  |  |

Notes. Significant values in bold. OR= Odds Ratio; CI= Confidence Interval

*Psychological symptoms were determined by dividing the sample into two halves (threshold: Median=14) based on the scores of the PGWB-S (36). The event considered was presenting psychological symptoms, which was defined as scoring below the median.

**Multivariable analysis
